# Patients’ Perspectives on the Quality and Safety of Intravenous Infusions: A Qualitative Study

**DOI:** 10.1177/2374373519843921

**Published:** 2019-04-30

**Authors:** Carly Wheeler, Dominic Furniss, Galal H Galal-Edeen, Ann Blandford, Bryony Dean Franklin

**Affiliations:** 1 Centre for Medication Safety and Service Quality, Pharmacy Department, Imperial College Healthcare NHS Trust, London, United Kingdom; 2 UCL Interaction Centre, London, United Kingdom; 3 Faculty of Computers and Information, Cairo University, Giza, Egypt; 4 Research Department of Practice and Policy, UCL School of Pharmacy, London, United Kingdom

**Keywords:** patient satisfaction, patient participation, qualitative methods, intravenous infusions, patient safety

## Abstract

**Background:**

The administration of medication or fluids via the intravenous route is a common intervention for many hospital inpatients. However, little research has explored the safety and quality of intravenous therapy from the patient’s perspective, despite the role of the patient in patient safety receiving increased attention in recent years.

**Objective:**

To explore patients’ perspectives on the perceived quality and safety of intravenous infusions and identify implications for practice.

**Method:**

Qualitative semistructured interviews were conducted with 35 hospital patients receiving intravenous infusions in critical care, oncology day care, general medicine, and general surgery areas within 4 National Health Service hospitals in England. Data were analyzed thematically.

**Results:**

Four underlying and interlinked themes were identified: knowledge about intravenous infusions, challenges associated with receiving intravenous infusions, the role of health-care professionals, and patients’ attitudes toward receiving infusions.

**Conclusions:**

Patients were generally satisfied with receiving infusions; however, factors that contributed to decreased feelings of quality and safety were identified, suggesting areas for intervention. Issues to do with infusion pump alarms, reduced mobility, cannulation, and personal preferences for information, if given more attention, may improve patients’ experiences of receiving intravenous infusions.

## Introduction

The administration of medication or fluids via the intravenous route is a common intervention for many hospital inpatients. However, providing intravenous therapy is complex and data suggest that errors are common (1,2). Historically, research regarding intravenous therapy has tended to focus on identifying the causes of these errors (3,4) and, more recently, testing interventions to reduce them (5). However, little research has studied the quality and safety of intravenous therapy from the patient’s perspective, despite the role of the patient in patient safety receiving increased attention in recent years. Patients can offer insights into the quality and safety of care, based on their own experiences, and provide details not captured by staff, which have the potential to inform policy and practice. Additionally, the role of patients’ involvement in their care has also been highlighted as part of the patient experience (6).

A workshop with 9 people with previous experience of receiving intravenous therapy, undertaken to inform the design of our wider program of work, highlighted some factors that influence the quality of patients’ experiences when receiving intravenous therapy (7). However, workshop participants were self-selected and their experiences of intravenous therapy historical, which may collectively have resulted in somewhat slanted views and biases. Other patient experience research has tended to focus largely on the venous access aspect of the infusion process (8–11).

We therefore wanted to explore views on the perceived quality and safety of infusion therapy from the perspective of a wider range of hospital patients who were receiving infusions during their hospital stay and to identify implications for practice.

## Method

### Design and Setting

This study formed part of a larger project, Exploring the Current Landscape of Intravenous Infusion Practices and Error (“ECLIPSE”) (12).

We conducted semistructured interviews with adult patients at 4 different acute hospital organizations in England. Patients were recruited from the following clinical areas: critical care and oncology day care (organization 1), general medicine and general surgery (organization 2), general medicine and oncology day care (organization 3), and general surgery (organization 4). The 4 hospital organizations and the clinical areas studied were purposively selected from among the 16 studied in phase 1 of ECLIPSE (13) on the basis of trying to achieve maximum variation in geography, infusion pump use, and local practices.

### Data Collection

An interview topic guide (Supplementary Material) was developed based on relevant literature and the earlier patient involvement workshop (7) to explore patients’ views on issues and activities relating to the administration of intravenous infusions. Demographic data were collected in the form of patients’ year of birth and ethnic background. Ward nurses were asked to suggest adult patients who met the following criteria: receiving one or more intravenous infusion, well enough to be interviewed, and English speaking. In consultation with participating organizations, it was agreed that it was not appropriate to interview pediatric patients about their intravenous infusions and that it would be impractical to arrange interviews with their carers.

Semistructured interviews were conducted at patients’ bedside by 1 of 2 interviewers, between November 2016 and December 2017. Interviews were audio-recorded where possible; otherwise detailed notes were taken. Interviews lasted approximately 20 to 30 minutes, except in critical care where they were 10 to 15 minutes long. Audio-recorded interviews were transcribed verbatim.

### Data Analysis

Data were analyzed using thematic analysis (14). We used a combination of deductive and inductive approaches, guided initially by the framework of the interview topic guide, but also allowing for the emergence of new codes and themes. Cross-checking of coding strategies and interpretation of data was undertaken for 4 interviews by a second researcher; no disagreements were identified. Data analysis was organized using NVivo version 11. Emerging findings were presented and discussed at a patient and public involvement workshop to ensure that themes were interpreted from a user perspective as well as a researcher perspective.

### Ethical Considerations

Ethical approval was obtained from a National Health Service Research Ethics Committee (14/SC/0290) and site-specific approval from each participating hospital organization. All participants provided written consent prior to interview and all data were anonymized prior to analysis.

## Results

### Participants

Thirty-five patients were interviewed. Patients had an age range of 27 to 89 years and were from the following ethnic backgrounds: white British/English (n = 28), Pakistani/Indian (n = 3), Pakistani (n = 2), Sri Lankan (n = 1) and black British (n = 1). Twenty were male and 15 female. We interviewed 10 patients from organization 1, 10 from organization 2, 10 from organization 3, and 5 from organization 4. Patients were in general medicine (n = 5), general surgery (n = 10), oncology day care (n = 12), critical care (n = 5), and an acute medical unit (n = 3). For 24 patients, the interview was audio-recorded, and for the remainder, detailed notes were taken.

Four themes emerged from the interviews relating to patients’ perspectives on the perceived quality and safety of intravenous infusions: knowledge about intravenous infusions, challenges associated with receiving intravenous infusions, the role of the health-care professional, and attitude toward intravenous infusions. These are described in turn.

### Knowledge About Their Intravenous Infusions

Patients discussed both the information they had received and how this information had been communicated to them. Most patients described having some general knowledge around their intravenous therapy, such as why they were receiving an infusion and the broad function of the infusion pump. However, there was wide variation in the depth of this knowledge, including the names of the medications that they were receiving and details of infusion pumps such as the causes of alarms. Despite varying levels of knowledge surrounding the infusion process, the majority of patients were satisfied with how informed they felt, highlighting variation among patients in terms of their desire for information. For some, feeling informed was very important for feeling reassured about their treatment, with a good infusion experience being one in which they felt they knew what was going on. Others were less interested and felt satisfied without knowing detailed information. In some instances, this was just a personal preference, but several acutely unwell patients described challenges that made the receipt of information difficult and reported feeling that receiving detailed information was low priority in the circumstances, as highlighted by the patient below when asked whether they were curious about what was going on:

No. And you know, I was just in so much pain. If these people were saying that this is going to work quicker, I was just go for it…I was told…I was given as much information as probably it would allow at that time. (Patient 1, general surgery, organization 4).

Patients who had a history of receiving infusions described needing less information now than they did the first time they received an infusion. One patient reported that receiving as little information as possible was her way of coping with her chemotherapy. A small number of patients described a desire for information so that they could be actively vigilant in their treatment process, with one patient who had previously had an allergic reaction wanting to check he was not receiving any medication he might be allergic to.

Verbal communication from nurses was the main way in which most patients received information about their infusions. Other sources of information included doctors, pharmacists, patients’ partners, other patients, leaflets, and the Internet. The majority of patients reported feeling comfortable asking questions if they did not receive all the information they wanted. For the small number of patients who did not feel comfortable asking questions or raising issues, all described previous negative experiences with requesting information:

I’d say I wasn’t really given much information about them [the infusions], when I did try to ask questions, so it seems as though I was not being taken notice of when I was asking. (Patient 3, general surgery, organization 4).

While patients largely spoke about receiving infusion information verbally, patients receiving planned infusions, particularly in oncology day centers, were more likely to have received written information. The idea of receiving an information leaflet, which could be read at a time convenient to the patient, was welcomed by many (but not all) patients in other clinical areas.

### Challenges Associated With the Infusion Process

When asked about the negative aspects of receiving intravenous infusions, 3 main challenges were highlighted. The most prominent was the nuisance of infusion pump alarms. Patients described alarms going off frequently and found this both annoying and disruptive, making sleep difficult for those who were inpatients. Some patients reported being able to identify the reason for alarms, particularly patients with a history of receiving infusions, citing blockages or backflows, routine warnings just before the infusion was due to finish, and then again when the infusion had actually finished. However, for those patients who had not received information about alarms, they were described as a cause for concern:

I think in the middle of the night when everything’s beeping you sort of panic a bit as a patient that something’s gone wrong or it means something serious. (Patient 10, critical care, organization 1)

Patients mentioned that it was not just their own alarms that were disruptive but also those of other patients nearby. Several patients had suggestions as to how the impact of alarms on patients could be reduced, including systems where alerts went directly to nurses, visual rather than audible alarms, and more patient involvement in monitoring and turning them off.

Maybe try a silent alarm type system for evenings. I just think it would make the whole ward more comfortable. You know, if you’ve got two or three going off…And the other trouble is, is they don’t all set them going at the same time, do they? So then you’ve got…One goes off, so then that gets sorted and then minutes later there’s another going, so…But, again, I’m not complaining about it. I just think there’s room for improvement. (Patient 10, acute medical unit, organization 3)

A second frequently mentioned challenge was the lack of mobility associated with receiving an intravenous infusion. The giving sets attached to the pump were viewed as restrictive, with even the smallest movement of the arm, such as using a mobile phone or reading a book, setting off the alarm particularly if using the antecubital fossa for venous access. Several patients spoke of having had their intravenous access points moved to their wrist or nondominant arm and that this had made a big improvement in terms of not having to keep still:

That is something that for some people their stronger arm is so strong that it’s almost…they’re almost kind of imprisoned if the cannula is on that arm. (Patient 5, general surgery, organization 4)

The limiting nature of being attached to an intravenous infusion had affected several patients’ sleep, with some describing having become tangled in the giving set. Being unable to freely walk while attached to an infusion was also frustrating for many, even though for the majority of infusions it is possible to request to be detached briefly, for example, to visit the toilet.

A third challenge discussed, particularly by patients with a history of receiving infusions, was pain and discomfort relating to cannulation. Within all clinical areas, there were patients who spoke of having poor or deep veins, which had made the process of cannulation difficult and resulted in cannulas having to be resited:

The worst part is the cannula. The cannula is difficult, because after a while your veins get used so much that they can’t find the vein and it’s very difficult for them as well. I’ve had to have mine in here, in the side of my hand, because my veins are non-existent now. (Patient 5, oncology day care, organization 1).

### Health-Care Professionals’ Behavior

The health-care professionals that patients encountered during their treatment, in particular nurses, were frequently discussed alongside descriptions of their infusion experience. Instances where staff were approachable and put patients at ease through talking with them made the experience a positive one and this was largely the picture that patients painted. Frequent checking of infusion equipment by staff was also felt to be reassuring, as was checking of patient identification wristbands and staff adherence to good hygiene practices, such as wiping equipment and wearing gloves. A high level of trust in staff was reported and offered as an explanation by some as to why they did not feel they needed to know everything about the infusion process:

I’ve got an interest in what I’m doing, but, you know, I don’t want to replicate the doctors, I don’t want to be second guessing the doctors or particularly checking up on them. I trust them. (Patient 8, oncology day care, organization 3)

Additionally, several patients reported being happy to receive or have their infusions changed while they were sleeping or while they were unable to see what was happening due to limited mobility. Patients also used language reflecting their feelings toward staff including “expert” and “professional.” This was demonstrated when patients were asked whether they had ever interacted with their infusion pumps themselves; most had not and reported leaving it to the health-care professionals. Conversely, there were a small number of incidents where staff behavior had left patients feeling unsafe. In one instance, during a previous cycle of chemotherapy, a patient had been mistaken for another patient by a member of staff; she and her partner had subsequently been extra vigilant in checking her treatment. Descriptions were provided by several patients where they felt nurses were lacking knowledge on how to use infusion pumps, which they found disconcerting.

In terms of quality of care, patients in oncology units appreciated the continuity of seeing the same nurse and the individual attention paid to them, with the opposite described on larger inpatient wards, as the patient below contrasted with their current, more attentive, critical care experience:

I mean I know they keep an eye and everything else, but in a bigger ward you know, you may get a time element where you won’t see anybody. (Patient 6, critical care, organization 1).

Additionally, one inpatient perceived a marked difference in the quality of care received between days and nights, with more staff and support during the day. This was evidenced when his cannula fell out during the night and could not be reinserted until the morning, which he perceived to be due to reduced staff numbers and therefore increased workload during the night shift.

### Attitude Toward Receiving Intravenous Infusions

For the most part, patients were satisfied with their experiences of receiving intravenous infusions. In particular, patients widely described an appreciation for the care they received. While the occasional negative experience was described, this was often within the context of the patient describing the health-care setting as being busy with overworked staff. Patients accepted negative experiences within the challenges of the setting.

This is more relaxed than on the ward, but then they’ve got a lot more people to look after on the ward. They’ve got to look after everyone, haven’t they? Whereas here, you just have your chemo and they come in and connect you up and then when one runs out they come and connect you up with the other one. So. They’re not as, they don’t seem as rushed I suppose. (Patient 1, oncology day care, organization 1).

Linked to this, patients across all settings approached their infusions with a “just get on with it” attitude. Patients recognized that the treatment they were receiving was necessary and accepted what this entailed. This attitude was illustrated by one patient when asked whether anything could be changed to improve the infusion process:

I don’t think so because I know it’s got to happen. Just looking forward to it coming out really. (Patient 9, critical care, organization 1)

As noted earlier, patients generally assumed a passive role in the infusion process, with health-care professionals in control.

## Discussion

This study explored 35 patients’ perspectives on the perceived quality and safety of intravenous infusions. Four main interlinked themes emerged highlighting patients’ experiences, both positive and negative, of receiving infusions. Patients generally reported positive experiences of receiving IV infusions, feeling satisfied with the amount of information received and health-care professionals’ behavior. Patients approached their intravenous infusions in a very matter-of-fact way, accepting that they might include undesirable aspects. Nevertheless, this study did identify aspects of infusion treatment which, if addressed, may improve patient experience.

As found in earlier research, pain associated with cannulation was described as an unpleasant experience (9,10). In line with previous work, this study also highlighted that pump alarms can be a significant source of patient concern (7) and cause discomfort in preventing patients from sleeping (9). As has been widely reported in health-care research (15), the role of the health-care professional, in this study primarily nurses, was very important in providing a positive patient experience. Additionally, the attitude of health-care professionals was a key factor in patients’ willingness to ask questions (16). Those who reported having previously experienced an error or adverse event were more likely to report being involved in checking and less likely to be passive.

Uniquely, this study also highlighted the different information needs of patients depending on their level of experience with intravenous infusions, with patients with chronic conditions more knowledgeable about their treatment and hospitals compared to first-time patients. The acuteness of patients’ conditions was another factor influencing the receipt of information, with different modes and times of delivery felt by patients to be appropriate, depending on their ability to take in and process information. The difference in patient experience in receiving intravenous infusions in different settings and clinical areas was also revealed.

### Implications

In terms of implications for health-care practice, findings indicate the importance of individual patient preferences in ensuring a positive patient experience. There are potential benefits of ensuring patients are informed and involved with their infusion therapy to the extent that they wish to be, and from recognizing the patient as a key source of information about their previous treatments and any associated problems. Additionally, as suggested in the existing literature (9), practical changes to policy regarding cannula placement, aiming to avoid the dominant arm and the antecubital fossa if possible, may also be helpful.

In terms of further research, the major issue highlighted related to infusion pump alarms; this warrants further study to identify ways of improving their design and configuration so as to reduce patient discomfort while maintaining clinicians’ situation awareness of the status of pumps and intravenous infusions. As this study focused on adult patients, further research is also required to explore the perspectives of pediatric patients and their carers.

### Strengths and Limitations

A strength of this study is that we interviewed a large number of patients in different clinical areas, including the relatively hard-to-reach clinical care population, across 4 different hospitals. To minimize recruitment bias, maximum variation sampling was undertaken; however, this was limited by the patients available at the time of interview and the challenge of finding patients who were well enough to participate. A limitation is that we considered only the perspectives of adult patients and not those of pediatric patients or their carers.

## Conclusion

We found that patients were generally satisfied with receiving intravenous infusions; however, factors that contributed to decreased feelings of quality and safety were identified, suggesting areas for intervention. Issues to do with infusion pump alarms, reduced mobility, cannulation, and personal preferences for information, if addressed, may improve patients’ experiences of receiving intravenous infusions and contribute to the safety and quality of patient care.

## Supplemental Material

Supplemental Material, Topic_Guide_Journal_of_patient_experience - Patients’ Perspectives on the Quality and Safety of Intravenous Infusions: A Qualitative Study Click here for additional data file. Supplemental Material, Topic_Guide_Journal_of_patient_experience for Patients’ Perspectives on the Quality and Safety of Intravenous Infusions: A Qualitative Study by Carly Wheeler, Dominic Furniss, Galal H Galal-Edeen, Ann Blandford and Bryony Dean Franklin in Journal of Patient Experience
