# Supplementary material for: Patients’ Perspectives on the Quality and Safety of Intravenous Infusions: A Qualitative Study
Source: J Patient Exp. 2019 Apr 30;7(3):380–5. doi: 10.1177/2374373519843921 (PMC7410139; doi:10.1177/2374373519843921)
Supplement: Supplemental Material, Topic_Guide_Journal_of_patient_experience - Patients’ Perspectives on the Quality and Safety of Intravenous Infusions: A Qualitative Study [file Topic_Guide_Journal_of_patient_experience.pdf]

ECLIPSE PHASE 2:
INTERVIEW GUIDE FOR INTERVIEWS WITH **PATIENTS**.
TOPIC GUIDE

INTRODUCTION

→ Introduction of interviewer, background and motivation for the study and what we'll talk about (scope).

The purpose of this interview is to explore your experience as a patient in receiving IV therapy. There are no right or wrong answers; the interview is simply about hearing your views on this topic and learning from your experience. This interview aims to give us more depth and understanding of practice and what this entails for you as a patient.

We will not use your name in any reports of this work and it will not be made known who took part. However, some of the things you say in the interviews might be used to illustrate and support the findings of the research. We will make every effort to make sure that these remain unidentifiable.

Are you happy for this interview to be tape recorded? Only researchers who are part of the team will have access to the recording and you will not be named in transcriptions of the interview.

Note: Interviews are planned to last for 30mins. **We might not have time for all of these questions so priority questions are underlined.**

CONTEXT

1. **Could you first tell me a little about your experience here as a patient?**

   Prompts:
   - How long have you been in hospital?
   - Have you been on this ward for long; how would you describe it?
   - How would you describe how you related to your IV infusion treatment? (e.g. inquisitive/uninterested, will speak up/won't speak up)

2. **Have you received many infusions as part of your treatment(s), either now or in the past?**

INTERACTION WITH STAFF

3. <u>**Could you talk me through what they do from your perspective in terms of IV treatment?**</u>

   Prompts:
   - Do all the staff administer IV infusions in the same way?
   - Do any of them do things differently that you like or don't like?
   - Could you describe a good time and a bad time?
   - Is there something you wish all of them would do, or perhaps they wouldn't do?

4. <u>**What information are you told, if any, about your infusions?**</u>

Prompts:

- Who gives you this information?
- Are you happy with this level of information?
- What information do you want?
- Do you feel like you understand enough about your infusions?

5. **What information about intravenous infusions do you think would be useful to provide other patients? What would be the best way to share this information (e.g. leaflet)?**

6. **Do you like to know what's going on with your infusions in detail, or would you prefer it if the healthcare staff just got on with it?**
(1 signifying you don't really want to know the detail and 10 signifying you really want to know what's going on) – why?

7. **How comfortable do you feel about raising questions or concerns about your infusions with staff?** (1 being not comfortable at all and 10 being completely comfortable) – why?

IV EXPERIENCE

8. **<u>Have you had any issues with how your infusions have been set-up?</u>**

Prompts:

- Or any issue with being on the infusions once they are set-up, day or night?
- Are there any issues with the pumps that are used?

9. **<u>Have you used your own infusion pump directly (e.g. pushed any buttons)?</u>**

Prompts:

- Have you seen/heard about other patients doing so?

10. **<u>What's the one thing you would do to change intravenous infusion practice for the better?</u>**

ROUND-UP

11. **Is there anything else that you think it would be useful to share on this topic?**
12. **Do you have any questions for me? Or any hopes or concerns for the project?**

Thank you very much for your time and your help with this study!
